# Genetic Structure, Diversity and Long Term Viability of a Medicinal Plant, *Nothapodytes nimmoniana* Graham. (Icacinaceae), in Protected and Non-Protected Areas in the Western Ghats Biodiversity Hotspot

**DOI:** 10.1371/journal.pone.0112769

**Published:** 2014-12-10

**Authors:** K. Nagaraju Shivaprakash, B. Thimmappa Ramesha, Ramanan Uma Shaanker, Selvadurai Dayanandan, Gudasalamani Ravikanth

**Affiliations:** 1 Department of Crop Physiology, University of Agricultural Sciences, Bangalore, Karnataka, India; 2 School of Ecology and Conservation, University of Agricultural Sciences, Bangalore, Karnataka, India; 3 Department of Biology and Centre for Structural and Functional Genomics, Concordia University, Montreal, Quebec, Canada; 4 Ashoka Trust for Research in Ecology and the Environment, Bangalore, Karnataka, India; 5 Québec Centre for Biodiversity Science, Montréal, Québec, Canada; CSIR- National institute of oceanography, India

## Abstract

**Background and Question:**

The harvesting of medicinal plants from wild sources is escalating in many parts of the world, compromising the long-term survival of natural populations of medicinally important plants and sustainability of sources of raw material to meet pharmaceutical industry needs. Although protected areas are considered to play a central role in conservation of plant genetic resources, the effectiveness of protected areas for maintaining medicinal plant populations subject to intense harvesting pressure remain largely unknown. We conducted genetic and demographic studies of *Nothapodytes nimmoniana* Graham, one of the extensively harvested medicinal plant species in the Western Ghats biodiversity hotspot, India to assess the effectiveness of protected areas in long-term maintenance of economically important plant species.

**Methodology/Principal Findings:**

The analysis of adults and seedlings of *N. nimmoniana* in four protected and four non-protected areas using 7 nuclear microsatellite loci revealed that populations that are distributed within protected areas are subject to lower levels of harvesting and maintain higher genetic diversity (H_e_ = 0.816, H_o_ = 0.607, A = 18.857) than populations in adjoining non-protected areas (H_e_ = 0.781, H_o_ = 0.511, A = 15.571). Furthermore, seedlings in protected areas had significantly higher observed heterozygosity (H_o_ = 0.630) and private alleles as compared to seedlings in adjoining non-protected areas (H_o_ = 0.426). Most populations revealed signatures of recent genetic bottleneck. The prediction of long-term maintenance of genetic diversity using BOTTLESIM indicated that current population sizes of the species are not sufficient to maintain 90% of present genetic diversity for next 100 years.

**Conclusions/Significance:**

Overall, these results highlight the need for establishing more protected areas encompassing a large number of adult plants in the Western Ghats to conserve genetic diversity of economically and medicinally important plant species.

## Introduction

The harvesting of medicinal plants from wild sources to meet pharmaceutical industry needs [1]–[3] may reduce populations of many plant species to below minimum viable population sizes, leading to eventual extinction of numerous medicinally important plant species [2], [4]. The long-term survival of these species will largely depend on the effectiveness of protected areas in sustaining viable populations that may serve as genetic stocks to aid replenishing dwindling populations in harvested areas [5]–[9]. Although protected areas may play a central role in conservation of biological diversity and genetic resources [10], [11], their effectiveness in preventing genetic erosion of many species remain largely unknown. Several studies have focused on assessing the effectiveness of protected areas in conserving genetic resources of Non Timber Forest Products (NTFP) in the Western Ghats of India [9], [12]–[15]. These studies have revealed that some plant species including bamboos and rattans harbor higher genetic diversity in protected areas than in non-protected areas or at peripheral regions of the protected areas [13]–[15]. However, the effectiveness of protected areas in conserving medicinal plants remains unknown. Comparative studies of medicinal plants in protected and non-protected areas provide ideal means to evaluate the effectiveness of protected areas in maintenance of genetic diversity and long-term viability of medicinally important plant populations [14].

**Figure 1:**
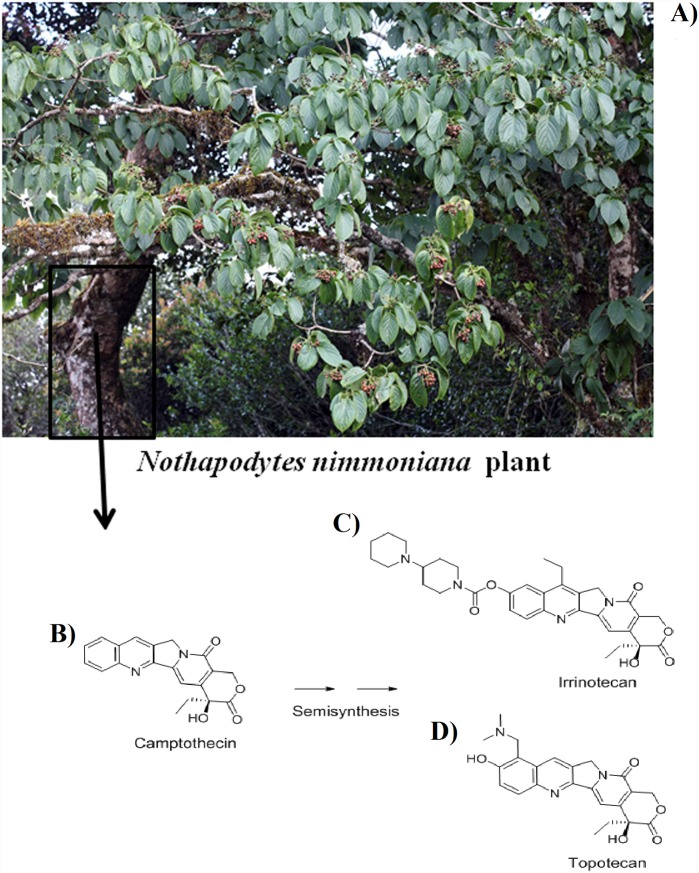
Image of A) *N. nimmoniana* plant and chemical structure of B) Camptothecine extracted from wood of *N. nimmoniana* C) Irinotecan and D) Topotecan, two clinically used drugs synthesized from Camptothecine as a precursor.

In recent years, *Nothapodytes nimmoniana* (Graham) Mabb., one of the medicinally important tree species distributed in the Western Ghats has become a major source of DNA topoisomerase inhibiting anti-cancer drug, Camptothecine (CPT) (Fig. 1), one of the alkaloids sought after by the pharmaceutical industries around the world [16]. The global demand for CPT exceeding an annual market value of over US$ 4 billion [17] led to a large-scale exploitation of the species from its wild habitats in the region resulting in an estimated loss of 20% of *N. nimmoniana* populations in the Western Ghats [18], [19]. Consequently, *N. nimmoniana* has been declared as an endangered/vulnerable plant species [18], [20].

We conducted genetic and demographic studies of *N. nimmoniana* populations in protected and non-protected areas in the central Western Ghats of southern India to assess the genetic and demographic effects of harvesting and evaluate the effectiveness of protected areas in the maintenance of long-term viability of *N. nimmoniana*. The specific objectives of our study were to 1) assess the genetic structure and diversity of *N. nimmoniana* populations in protected and non-protected areas, 2) investigate any evidence for genetic bottlenecks of populations and 3) analyze demographic data to predict future population sizes to evaluate long-term viability of *N. nimmoniana* populations in the Western Ghats.

## Materials and Methods

### Ethics Statement

The field work and tissue sample collection of *Nothopodytes nimmoniana* was carried out in the central Western Ghats regions of Karnataka, with permission from the Karnataka Forest Department. Tissue sampling was carried out under the supervision of forest officers and used solely for scientific research. The sampling was non-invasive with no impact on the natural growth or regeneration of *N. nimmoniana* populations in the wild.

### Study sites and design

**Figure 2:**
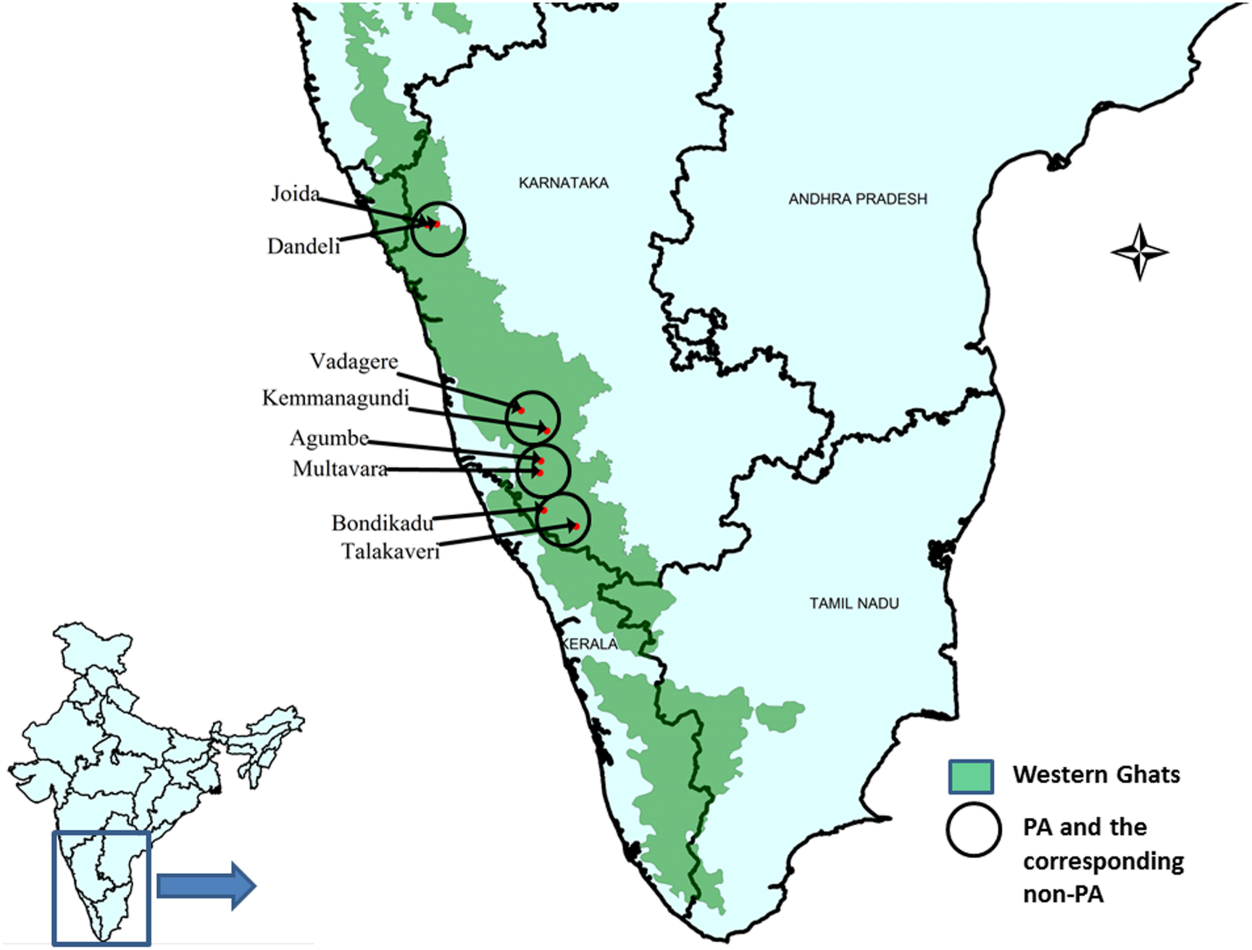
Map showing the distribution and sampling locations of *Nothapodytes nimmoniana* in the Western Ghats, India.

This study was conducted in the Western Ghats, India, one of the 32-biodiversity hotspots of the world [21]. The Western Ghats includes 34 national parks and wildlife sanctuaries covering an area of about 7300 km^2^ [22]. The distribution map of *N. nimmoniana* was overlaid with protected area map of the Western Ghats to identify populations that are distributed within protected areas (PAs), and based upon the availability of *N. nimmoniana* within the PA network of the Western Ghats, four study sites namely: Dandeli Wildlife Sanctuary (established in the year 1974), Talakaveri wild life sanctuary (established in the year 1987), Kudremukh wild life sanctuary (established in the year 1987) and Agumbe medicinal plants protected area (established in the year 1999) were selected for the present study (Fig. 2, Table 1). Similarly, four populations located adjacent to protected areas were also selected and treated as non-protected areas (NPAs) (Fig. 2, Table 1).

**Table 1:** Location, geographical coordinates, sample size (N) and private alleles (A_P_) in eight populations of *Nothapodytes nimmoniana*.

Population Name	Status	Latitude	Longitude	Sample size (N)		*A_P_*
				Adults	Seedlings	
Dandeli	PA	15°11′59.1″	74°35′59.1″	17	20	11
Talakaveri	PA	12°24′0″	75°30′0″	20	20	13
Kemmangudi	PA	13°20′59.1″	75°27′0″	13	8	6
Agumbe	PA	+−13°3′0″	75°5′27.6″	13	0	6
Joida	NPA	15°10′9.5″	74°29′4.2″	19	12	5
Multavara	NPA	14°22′11.1″	75°0′0″	11	9	6
Vadagere	NPA	12°26′59.1″	75°54′0″	13	0	3
Bondikadu	NPA	12°50′59.1″	75°50′59.1″	20	3	7
**Over all**	**PA+NPA**			**126**	**72**	**57**

Note: PA = Protected area, NPA = Non-protected area.

### Study species

*Nothapodytes nimmoniana* (Graham) Mabb. (Icacinaceae), (Fig. 1A) formerly known as *Nothapodytes foetida* (Wight) Sleumer and *Mappia foetida* Meirs is a medicinally important tree species naturally distributed in many parts of the Western Ghats in South India, some parts of Assam, Himalayan foothills, Sri Lanka, Burma and Thailand [19]. The bark of the stem is one of the richest sources of the anti-cancer compound, Camptothecine (Fig. 1B) [16], [23]. The two clinically used drugs, Irinotecan (Fig. 1C) and Topotecan (Fig. 1D), are currently semi-synthesized using natural Camptothecine as a precursor [24]. These trees are harvested by felling the trunk. Although stumps of cut trees often coppice, the continuous harvesting negatively impact the long term survival and reproduction. The extensive harvesting of *N. nimmoniana* led to severe reduction in population sizes [19] and currently classified as a ‘vulnerable/endangered’ species [18]. Although *N. nimmoniana* is a dioecious species, some individuals are polygamous with male, female and bisexual flowers [25].

### Population structure

In each of the four PAs and their adjoining non-PAs, 10 quadrats (10 m×10 m) were laid out randomly and data on the number of trees per quadrat, girth of all individuals above 10 cm dbh, and number of harvested or coppicing individuals were recorded. As a measure of regeneration, the number of seedlings and saplings (<1 m height) in each quadrat was recorded. For each quadrat, the number of regenerants (saplings and seedlings) was divided by the number of adults to obtain an index of the regeneration per adult. The number of harvested adults of *N. nimmoniana* in each quadrat was recorded and expressed as a percentage of the total number of adults harvested for each quadrat [11], [26]. The differences in the various parameters across PA and the non-PA were analyzed using Student’s *t*-test. The girth-class distributions of adult individuals in each site were determined. The frequency distribution of the girth-class of adults across the PA and non-PA was statistically evaluated using the nonparametric Kolmogorov–Smirnov test [27].

### Genetic diversity

#### DNA extraction and amplification protocol

Leaf samples were collected from 15–20 adults as well as seedlings selected randomly from four PAs and their adjoining Non-PAs. In each site, the area sampled was approximately 0.5 km^2^. Leaf samples were air-dried and preserved in silica gel until the extraction of total genomic DNA using a modified CTAB (cetryl trimethyl ammonium bromide) protocol [28] at the Conservation Genetics Laboratory at ATREE. The extracted DNA was visualized on ethidium bromide stained 0.8% agarose gels and quantified by measuring the absorption at 260 nm using a spectrophotometer. The DNA was diluted to a concentration of 10 ng/µl and used for PCR amplification. The seven microsatellite primer pairs [29], which gave consistent amplification with good level of polymorphism was selected for genotyping. The PCR amplifications for genotyping were carried out in an Eppendorf Mastercycler Gradient, (Eppendorf) thermal cycler. The PCR amplification was carried out in a 25-µl-volume reaction mixture containing 25 ng template DNA, 2.5 µl 10× reaction buffer containing 15 µM MgCl_2_, 3 µM of each dNTP, 0.25 µM each forward and reverse primer and 0.5 unit *Taq* DNA polymerase (Sigma). The thermal cycling parameters were: 94°C for 3 min, 35 cycles of 94°C for 40 s, 58–62°C for 40 s, 72°C for 60 s, followed by a final extension of 5 min at 72°C. The amplified samples were prepared for genotyping by mixing 10 µL of deionized formamide, 0.1 µL of 35–500 bp internal size standards (Tamara GeneScan-500, Applied Biosystems) and 1 µL of PCR product. The mixture was denatured at 95°C for 2 min and immediately placed on ice for a minimum of 5 min and electrophoresed on an ABI PRISM 310 Genetic Analyser (Applied Biosystems) to detect fragment sizes. The electrophorograms were analyzed with the GeneScan 3.7 and GenoTyper 3.7 software programs (Applied Biosystems).

### Data analysis

#### Microsatellite based genetic diversity measures

The following genetic diversity measures were calculated for all eight populations and also separately for adults and seedlings: Allelic distribution, private alleles (alleles unique to a population and not shared with other populations), the mean number of alleles per locus (averaged across 7 loci) and per population were calculated using GENEPOP 3.2a [30]. A standardized estimate of allelic richness per locus (averaged across 7 loci) and per population adjusted to the sample size [31]–[32] was calculated using the program FSTAT 2.9.3 [33]. ARLEQUIN version 3.1 [34] was used to test for heterozygote deficiency at each microsatellite locus for each population using a Hardy-Weinberg test based on Markov Chain iterations [35]. The pairwise test for linkage disequilibrium for each pair of loci for each allele in each population and Wrights F-statistics (Fis and Fst) were calculated using the program GENEPOP 3.2a [30].

#### Population genetic structure

We calculated the total genetic differentiation among population as Fst: which assumes an Infinite Allele Model (IAM) [36] by computing theta (θ) [37] and Rst: which assumes a Stepwise Mutation Model (SMM) [38] by computing Rho which adjusts for differences in sample size and allele size variances among loci using the software program FSTAT version 2.9.32 [33]. The proportions of genetic variation partitioned among populations and among groups of populations [39] were quantified using analyses of molecular variance (AMOVA) as implemented in ARLEQUIN V3.1 [34], and the statistical significance was tested with 10000 permutations. A model based Bayesian clustering method as implemented in STRUCTURE version 2.3.3 [40] was used as an alternative approach to examine the spatial genetic structure. The programme was run without prior population information under the admixture model (individuals may have mixed ancestry) and correlated allele frequency. Length of the burn-in was 100 000 and the number of MCMC replications after the burn-in was 1 000 000. Twenty independent chains were run for each K from K = 1 to K = 15. The method of Evanno et al [41] was used to find the most likely value of K by plotting log probability (L(K)) and ΔK of the data over multiple runs and as implemented in STRUCTURE HARVESTER [42]. CLUMPP v.1.1.1 [43] software (resolves the label switching and compute average admixture co-efficient) was used to align the repetitions for each K, using G’ (10 000 repeats). The output from CLUMPP was used for ancestry analysis.

### Population bottleneck test and simulation of loss of diversity

We assumed that, if extensive harvesting of trees in recent years has affected the populations, they should show signatures of recent genetic bottlenecks between adults and seedlings. We also expect that, the populations outside the PAs should experience higher signatures of genetic bottleneck than populations within PA network, as populations within PAs receive more protection and less harvesting than NPAs. We used following approaches to test these predictions: Populations that experienced a bottleneck often exhibit a reduction of allele number and heterozygosity at polymorphic loci, with allele numbers being reduced at a higher rate than heterozygosity [44], [45]. Thus, observed heterozygosity is higher than that expected based on allele numbers assuming mutation drift equilibrium [46]. We tested for a population bottleneck using BOTTLENECK version 1.2.0.2 [46], [47], which is based on the assumption that populations that may have undergone severe size reductions will show an excess of heterozygotes relative to allelic diversity. The BOTTLENECK analysis was run using the stepwise mutation model (SMM). The significance of genetic diversity excess (*H*_e_>*H*_eq_) was tested using Wilcoxon signed-rank tests and sign test [46] based on 5000 replications. A population bottleneck is also expected to change the allele frequency distribution [46], which could be detected as a shift in the mode of the allele frequency distribution. The mode-shift indicator test was performed to test [46], if the allele frequency distribution pattern shows a departure from approximately L-shaped (as expected under HWE) distribution.

To assess whether the current population size of *N. nimmoniana* is sufficient to maintain 90% of present day observed genetic variation over the next 100 years as a measure of long-term genetic viability of the species [44], we simulated future population parameters using the software program BOTTLESIM version 2.6 [48]. Based upon the present genetic diversity and population size, BOTTLESIM simulates future population genetic parameters (observed number of alleles [OA]) under different population bottleneck scenarios. Genetic diversity estimates over 100 years were simulated when retaining 100, 90, 75, 50, and 25 percent of the current population size. We performed 1,000 iterations with constant life history parameters (lifespan = 50 years, age at maturity = 10 years, completely overlapping generations, random mating, dioecious reproduction, and sex ratio of F: M: 1∶1).

## Results

### Population structure and harvesting pressure

**Figure 3:**
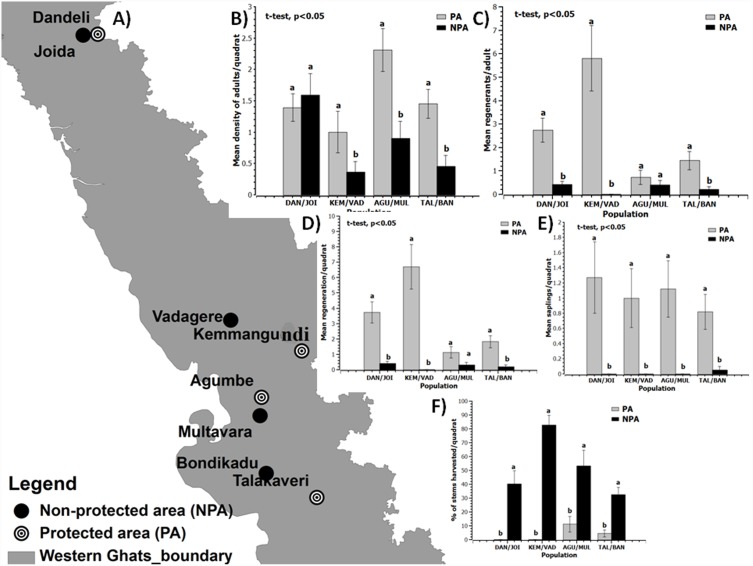
Demographic parameters of *Nothapodytes nimmoniana* in four protected and adjacent non-protected areas in the central Western Ghats, India. A) Western Ghats map showing sampling locations B) Mean density of adults per quadrat C) Mean regenerants per adult D) Mean regeneration per quadrat E) Mean saplings per quadrat F) Harvesting index. Note: Error bars are standard error. For each parameter, dissimilar letters above the bars are significantly different at 0.05 levels (t-test). PA, protected area; NPA, non-protected area; WG, Western Ghats; KEM, Kemmangundi; MUL, Multavara; TALA, Talakaveri; BAN, Bondikadu; DAN, Dandeli; JOID, Joida; AGU, Agumbe; VADE, Vadagere.

The mean density of adults between populations of PAs and NPAs were significantly different (t-test, P<0.05; Fig. 3B, S1 Table in S1 File). In general, populations in PAs showed higher mean density of adults than populations in the adjoining NPAs. The measure of reproductive turnover (mean regenerants per adult) was also higher in populations of the protected areas (t-test, P<0.05; Fig. 3C). We also found significant difference in overall proportion of reproductive individuals between PA and NPA populations (t-test, p<0.05, S1 Table in S1 File), with higher regeneration per quadrat and mean number of saplings per quadrat from populations of PA (Fig. 3D and 3E). The percentage of harvested adults per quadrat was higher in populations from NPA (t-test, p<0.05; Fig. 3F) than PA populations. Generally we observed that more adults were harvested (>40% of adults) from population of NPA and less than 5% adults were harvested from populations of PA (Fig. 3F) indicating *N. nimmoniana* populations outside PA are experiencing more threat and harvesting pressure.

### Genetic diversity and population genetic structure

**Figure 4:**
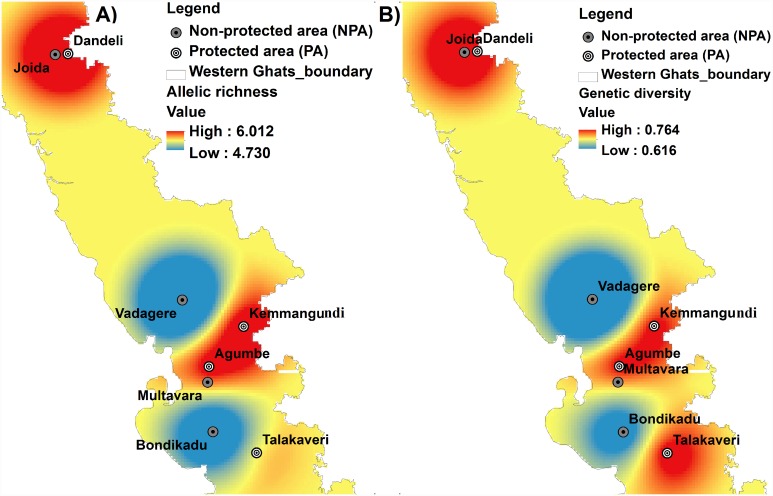
Map showing genetic diversity parameters at sampling locations of *Nothapodytes nimmoniana* from Western Ghats, India A) Nei’s gene diversity B) Allelic richness. Note: Regions represented in dark red indicate areas of high genetic diversity and allelic richness.

All seven-microsatellite loci were polymorphic in all studied populations. A total of 156 alleles with an average of 22.29 alleles per locus (A) were found at the species level (Table 2). The mean H_E_ and H_O_ across all loci were 0.805 and 0.567 respectively (Table 2). The mean expected heterozygosity (*HE*) was generally higher than mean observed heterozygosity (*Ho*) across all loci (Table 3). The locus NNM6a showed highest level of polymorphism with 5–18 alleles per population and expected heterozygosity ranged from 0.576–0.915 followed by the locus NN46 (A = 5–16 and H_e_ = 0.725–0.910). The locus NN54 had lowest level of polymorphism with 3–8 alleles per population and expected heterozygosity (H_E_) ranged from 0.394–0.779 followed by the locus NNT4 (A = 3–10 and H_E_ = 0.504–0.790). The allelic diversity parameters (allelic richness (A_R_) and mean observed number of alleles (A) per locus (averaged over 7 loci) were highest for the population Dandeli (A_R_ = 6.018 and A = 11.857±4.180) followed by Talakaveri (A_R_ = 5.476 and A = 10.714±4.309), Joida (A_R_ = 5.999 and A = 10.286±3.817) and the lowest values were found in populations Bondikadu (A_R_ = 4.974 and A = 8.143±4.298) and Vadagere (A_R_ = 4.727 and A = 8.571±2.226) (Table 3 and Fig. 4A). Many alleles were exclusive to specific populations, and these alleles were referred to as private alleles (A_P_). The two of the populations, namely Dandeli and Talakaveri had highest number of private alleles with 11 and 13 private alleles respectively (Table 1). The Nei’s gene diversity across all loci was highest in Joida (0.7633±0.087) and Dandeli (0.7461±0.102) populations. The lowest gene diversity values were observed in Vadagere (0.6158±0.192) and Bondikadu (0.6714±0.134) (Fig. 4B).

**Table 2:** Summary of genetic variation at seven microsatellite loci scored for eight *Nothapodytes nimmoniana* populations in the Western Ghats, India: expected and observed heterozygosity (*H_O_* and *H_E_*), and observed number of alleles per polymorphic locus (A), Weir & Cockerham (1984) estimates of *F_IT_*, *F_ST_*, *F_IS_* and *R_ST_*.

Locus	*H_O_*	*H_E_*	*A*	*F_IS_*	*F_IT_*	*F_ST_*	R_ST_
**NN54**	0.592*	0.757	12	0.098	0.215	0.130	0.113
**NNM14**	0.489*	0.798	22	0.342	0.409	0.102	0.124
**NNM6a**	0.429*	0.925	39	0.489	0.560	0.139	0.154
**Nn46**	0.642*	0.849	31	0.200	0.250	0.062	0.067
**Nn52**	0.659*	0.694	18	0.016	0.127	0.112	0.003
**NNT4**	0.546*	0.751	13	0.182	0.276	0.115	0.045
**NN50**	0.612*	0.859	21	0.234	0.295	0.079	0.098
**Over all loci**	**0.567** *	**0.805**	**22.286**	**0.233**	**0.313**	**0.105**	**0.096**

*indicates significant deviations of *H_E_* from HWE (at 0.05 significance level).

**Table 3:** Summary of genetic diversity parameters of *Nothapodytes nimmoniana* in four protected (PA) and adjacent non-protected areas (NPAs) in the central Western Ghats, India.

Populationgeneticparameter	Protectionstatus	Sampletype	KEM/VAD(Mean±SD)	TALA/BON(Mean±SD)	AGU/MUL(Mean±SD)	DAND/JOI(Mean±SD)	all PA/NPA(Mean±SD)
A	PA	Adults	6.714 (1.799)	8.857 (3.625)	8.000 (2.449)	7.429 (2.149)	15.286 (5.28)
		Seedlings	5.714 (1.976)	8.143 (2.160)	-	9.714 (3.352)	14.429 (6.10)
		Overall	8.571 (2.225)	10.714 (4.309)	8.000 (2.449)	11.857 (4.180)	18.857 (8.72)
	NPA	Adults	8.571 (2.22)	8.143 (4.298)	6.857 (2.478)	9.000 (2.64)**^a^**	15.571 (6.26)**^a^**
		Seedlings	-	-	5.143 (1.773)	5.71 (2.936)**^b^**	8.860 (4.53)**^b^**
		Overall	8.571 (2.226)	8.143 (4.298)	8.429 (2.226)	10.286 (3.817)	15.571 (6.26)
H_0_	PA	Adults	0.308 (0.133)	0.593 (0.162)	0.495 (0.188)	0.411 (0.165)	0.592 (0.12)**^a^**
		Seedlings	0.357 (0.222)	0.600 (0.197)	-	0.632 (0.239)	0.630 (0.087)**^a^**
		Overall	0.326 (0.144)	0.696 (0.162)**^a^**	0.495 (0.188)**^a^**	0.524 (0.194)	0.607 (0.100)**^a^**
	NPA	Adults	0.327 (0.144)	0.360 (0.220)	0.481 (0.146)**^a^**	0.556 (0.143)**^b^**	0.490 (0.11)**^b^**
		Seedlings	-	-	0.349 (0.268)**^b^**	0.309 (0.214)**^c^**	0.426 (0.200)**^b^**
		Overall	0.327 (0.144)**^b^**	0.360 (0.220)**^b^**	0.421 (0.173)	0.461 (0.170)	0.511 (0.111)**^b^**
H_E_	PA	Adults	0.710 (0.102)	0.798 (0.105)	0.807 (0.096)	0.782 (0.077)	0.829 (0.057)
		Seedlings	0.696 (0.197)	0.742 (0.098)	-	0.802 (0.075)	0.798 (0.075)
		Overall	0.739 (0.125)	0.775 (0.098)	-	0.806 (0.069)	0.816 (0.006)
	NPA	Adults	0.739 (0.124)	0.717 (0.140)	0.793 (0.034)	0.785 (0.097)	0.511 (0.114)
		Seedlings	-	-	0.645 (0.251)	0.751 (0.133)	0.783 (0.085)
		Overall	-	-	0.746 (0.071)	0.789 (0.082)	0.781 (0.094)
F_IS_	PA	Adults	0.397	0.342	0.397	0.297	0.209 (0.385)
		Seedlings	0.599	0.504	-	0.217	0.205 (0.327)**^a^**
		Overall	0.564	0.233	0.397	0.353	0.207 (0.358)**^a^**
	NPA	Adults	0.342	0.503	0.577	0.502	0.236 (0.346)**^a^**
		Seedlings	-	-	0.474	0.195	0.452 (0.325)**^b^**
		Overall	0.342	0.503	0.441	0.420	0.304 (0.350)**^b^**
F_ST_	PA	Adults	0.102	0.084	0.072	0.091	0.088 (0.010)
		Seedlings	0.090	0.084	-	0.091	0.087 (0.026)
		Overall	0.087	0.083	-	0.077	0.088 (0.029)
	NPA	Adults	0.099	0.093	0.082	0.067	0.092 (0.033)
		Seedlings	-	-	0.072	0.078	0.075 (0.029)
		Overall	-	-	0.078	0.065	0.088 (0.033)

Note: A = mean observed number of alleles, H_O_ = observed heterozygosity, H_E_ = expected heterozygosity, F_IS_ and F_ST_ = population specific fixation indices, − = data not present. Disimilar letters (a and b) above the parameter values indicates t-test significant at p<0.05.

**Figure 5:**
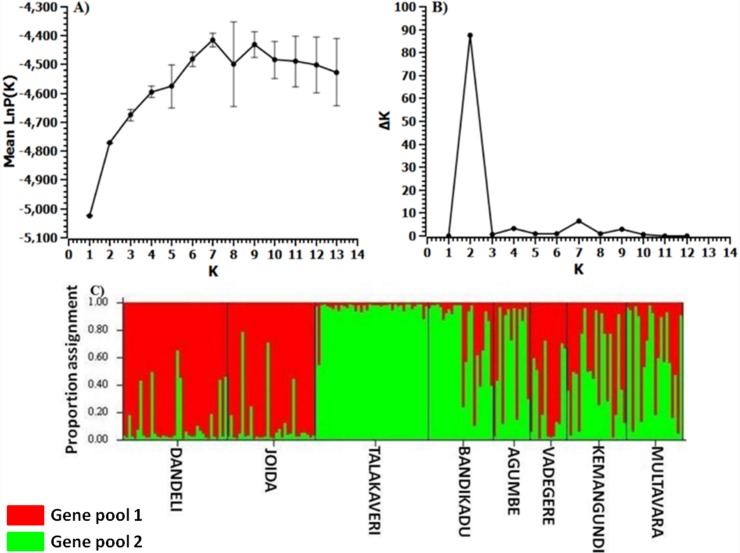
Results of Bayesian model based clustering method (STRUCTURE) analysis. a) Posterior probability of the data LnP(D) (±SD) against the number of K clusters, and increase of LnP(D) given K, calculated as (LnP(D)k–LnP(D)k–1) b) Delta K values from the mean log-likelihood probabilities from STRUCTURE runs where inferred clusters (K) ranged from 1 to 14 (c) Estimates of the proportion of ancestry, *Q*, in each of *K = 2* clusters for 198 *Nothapodytes nimmoniana* individuals using the model-based cluster analysis, STRUCTURE (version 2.1). Note: A single vertical line represents each individual with estimated membership in each cluster denoted by the different *colors*. The analysis was based on seven SSR loci and used admixture model of ancestry. Individuals are separated based on their population and black vertical lines in the bar chart are population identifiers. Populations are ordered as per their population name.

The F_ST_ estimate, *θ* averaged over seven loci was 0.105 (Table 2). The F_ST_ values between populations ranged from 0.065 to 0.099 (Table 3), indicating a low but significant level of genetic differentiation of populations. The overall F_IS_ and F_IT_ values were 0.233 and 0.313 respectively indicating loss of heterozygosity both at the population and at meta-population levels (Table 2). The F_IS_ values among populations ranged between 0.233 and 0.564. All populations had positive values of inbreeding (Table 3). The results of AMOVA indicated that most of the variations were within individuals (56.4%) followed by within and among populations (Table 4). The Bayesian clustering method revealed two optimum numbers of genetic clusters (K = 2; Fig. 5A, 5B and 5C) and geographically close populations grouped together corresponding to two genetically distinct clusters (Fig. 5C and Table 5) further supporting significant level of differentiation and genetic structure among populations.

**Table 4:** Analysis of molecular variance (AMOVA) for 195 individuals of *Nothapodytes nimmoniana* in protected (PA) and non-protected (NPA) areas.

Source of variation	d.f.	Sum ofsquares	Variance	% of totalvariance	F-statistics	p-value
Among groups (PA*NPA)	1	8.167	−0.057	−1.96	FCT = −0.012	0.973
Among populationswithin groups	6	102.886	0.284	9.72	FSC = 0.095	0.000
Among individualswithin populations	189	707.686	1.056	35.87	FIS = 0.389	0.000
Within individuals	197	324.5	1.647	56.35	FIT = 0.436	0.000
**Total**	393	1143.239	2.923			

**Table 5:** Membership of each pre-defined population in each of the two (K = 2) clusters generated by CLUMPP based on the results of STRUCTURE v2.2 analysis of SSR data.

Population name	*Q* value (proportion of individual ancestry In each cluster) K = 2	
	Cluster 1	Cluster 2
Dandeli	**0.886 (0.176)**	0.114 (0.176)
Joida	**0.895 (0.194)**	0.105 (0.194)
Talakaveri	0.040 (0.071)	**0.960 (0.071)**
Bondikadu	0.209 (0.272)	**0.791 (0.272)**
Agumbe	0.359 (0.377)	**0.641 (0.377)**
Vadagere	**0.710 (0.296)**	0.290 (0.296)
Kemmangundi	**0.515 (0.318)**	0.485 (0.318)
Multavara	0.376 (0.343)	**0.624** (0.343)
**Over all**	**0.501 (0.409)**	**0.499 (0.409)**

Populations contributing >50% of their ancestry to a single cluster are highlighted in bold.

### Genetic diversity in adults and seedlings

The comparison of genetic diversity parameters between adults and seedlings at the overall population level revealed no significant differences (A, H_O_ and H_E_). However, the comparison of genetic diversity parameters between adults and seedlings within individual populations revealed significant decrease in genetic diversity parameters (A and H_O_) in seedlings as compared to adults in some of the populations (Table 3). Both adults and seedlings showed positive values of F_IS_ (Table 3).

#### Effect of harvesting on genetic diversity

Genetic diversity varied between populations from PA and NPA sites. Observed heterozygosity (H_O_) for populations of PA was 0.607, significantly higher than (P<0.05) the 0.511 in populations of NPA. Populations from PA also had highest number of private alleles (Table 1). There was no significant difference in mean H_E_ and observed number of alleles, indicating that differences among PA and NPA populations are due to loss of rare alleles resulting from genetic bottleneck and genetic drift. There was also a significant difference in genetic diversity measures between life history stages (adults and seedlings) from PA and NPA’s. We observed that the adults and seedlings from PA’s had significantly (P<0.05) higher observed number of alleles (A) and highest observed heterozygosity compared to NPA populations (Table 3). We also found significant decrease in genetic diversity measures (A, H_E_, H_O_) from adults to seedlings (Table 3) in populations of NPA indicating populations from NPA experience loss of diversity due to harvesting pressure than PAs. The AMOVA results showed that most molecular variance was found within individuals (56.4%) of *N. nimmoniana* followed by among individuals within populations (36.4%) and among individuals within groups (9.2%) (Table 4). The variance among groups (PA and NPA) was very low and negative (−2%). The recorded F_SC_ and F_IT_ values of 0.095 and 0.436 indicate a moderate level of spatial isolation. Finally, populations from NPAs showed significantly high level of inbreeding than PA populations and inbreeding level in seedlings were higher than in adults (Table 3) indicating a decrease in effective population sizes in NPAs.

### The population bottleneck test and prediction of future population size through simulations

The population bottleneck analyses of individual populations with adults and seedlings combined together detected evidence for recent population bottlenecks in five out of eight populations (Wilcoxon’s and Sign test; P>0.05 and 0.01) indicating a deviation from mutation drift equilibrium (Table 6). However, mode shift test did not reveal distortion in allele frequency as compared to normal L-shaped distribution. The population bottleneck analysis between generations (adults and seedlings) showed that out of five populations examined, seedlings from two populations and adults from four populations exhibited excess or deficiency of heterozygotes (Wilcoxon sign rank test (0.05>p<0.01) and sign test (0.05>p<0.01) (Table 6) suggesting a deviation from mutation drift equilibrium. However, allele frequency distribution revealed a normal L-shaped distribution in both adults and seedlings suggesting mutation drift equilibrium. Irrespective of protected status, populations of both PA and NPA showed evidence of recent bottleneck in *N. nimmoniana*.

**Table 6:** Analysis of historical and recent genetic bottleneck based on stepwise mutation model (SMM) of microsatellite evolution and Mode shift test for allele frequency distribution.

Population Name	Sign test (two tailed p-value)			Wilcoxon signed rank test (two tailed p-value		
	SMM			SMM		
	A	S	A+S	A	S	A+S
**Dandeli (PA)**	0.002**	0.022*	0.002**	0.008**	0.016*	0.008**
**Joida (NPA)**	0.020*	0.616	0.113	0.055*	0.937	0.040*
**Talakaveri (PA)**	0.124	0.022*	0.002**	0.375	0.015*	0.008**
**Bondikadu (NPA)**	0.291	0.088	0.020*	0.110	0.078	0.020*
**Agumbe (PA)**	0.022*	-	0.020*	0.040*	-	0.039*
**Vadagere (NPA)**	0.020*	-	0.021*	0.040*	-	0.040*
**Kemmangundi (PA)**	0.650	0.102	0.289	0.578	0.297	0.297
**Multavara (NPA)**	0.248	-	0.097	0.375	-	0.039*

Note: – = data absent. *P, <0.05; **P, <0.01. A = adults, S = seedlings.

The significance of gene diversity excess (He. Heq) is an indication of recent effective population size reductions (bottlenecks). The significance was tested using Sign test and Wilcoxon signed ranks test [44] based on 5000 replications.

**Figure 6:**
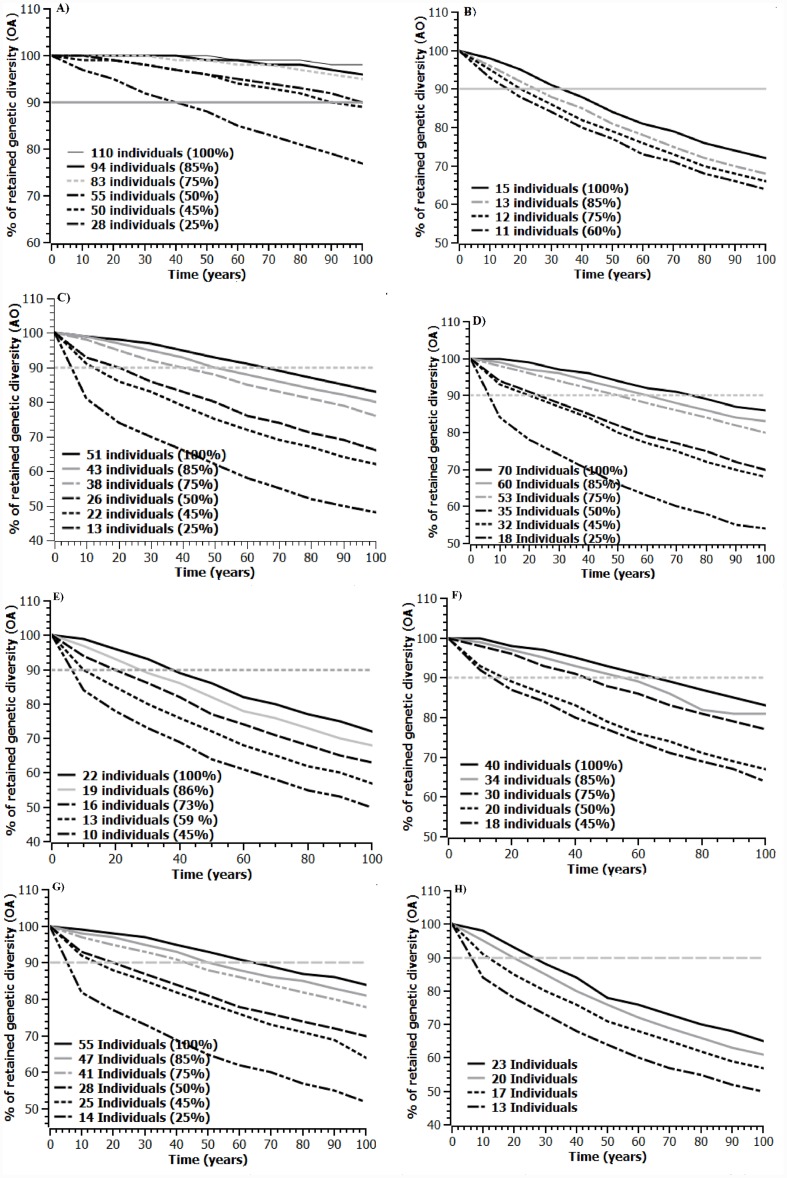
Predicted genetic diversity in eight populations of *Nothapodytes nimmoniana* sampled from protected (PA) and non-protected (NPA) areas of Western Ghats, India over next 100 years using BOTTLESIM. The current population size is unable to maintain 90% of current genetic diversity over the next 100 years in almost all sampled populations. A) Agumbe (PA) B)Vadagere (NPA) C)Dandeli (PA) D) Joida (NPA) E) Kemmangundi (PA) F) Multavara (NPA) G) Talakaveri (PA) and H) Bondikadu (NPA). Note: The observed numbers of alleles (OA) were projected to decline (Sex ratio 1∶1 F:M).

The BOTTLESIM based prediction of future genetic diversity with varying starting population sizes, and assuming current population size as the maximum (100%), the projected genetic diversity continued to decrease over time (Fig. 6). The observed allele diversity is expected to decrease at a higher rate than observed heterozygosity. With the exception of Agumbe population [for both PA and NPA] all other populations showed decline of genetic diversity beyond 90% of current genetic diversity values even at the retention of 100% of individuals (Fig. 6A to H). The populations in NPA are predicted to lose allelic diversity faster than PA populations. The overall results clearly showed that current population size of *N. nimmoniana* in Western Ghats is not sufficient to maintain present levels of genetic diversity for the next 100 years.

## Discussion

### Genetic diversity

At the species level, pattern of genetic diversity observed within *N. nimmoniana* is similar to other tropical tree species with similar life history and ecological traits [49]–[54] but higher than other tropical tree species with different life history or ecological traits [55]–[57]. Although populations are sparse and patchy, *N. nimmoniana* is widely distributed throughout Western Ghats in peninsular India and expected to maintain moderate to high level of genetic diversity. The mating system of *N. nimmoniana* is mixed, which consists predominantly outcrossing mating system with some selfing [19], [58] and seeds are dispersed by small birds [59]. The genetic diversity in *N. nimmoniana* (A = 22.28, A_R_ = 4.108, H_O_ = 0.567, H_E_ = 0.805) is comparable to other tropical tree species with mixed mating system and bird dispersed seeds [50], [52], [54]. However, the genetic diversity values of *N. nimmoniana* are lower than those reported in other microsatellite based studies of tropical tree species [49], [51], [60]–[62].

The comparison of observed (H_O_) and expected (H_E_) heterozygosity levels within populations of *N. nimmoniana* showed significant excess of homozygosity. This could be attributable to non-random mating with high gene exchange among related individuals and selfing due to the mixed mating system of the species. It is possible that reduction in population size due to habitat fragmentation or harvesting may have increased selfing rates and/or mating among closely related individuals resulting in a higher proportion of homozygous individuals in populations.

### Genetic structure

The values of inter-population genetic differentiation based on both measures were relatively low (F_ST_ = 0.105 R_ST_ = 0.096), similar to each other and significantly different from zero suggesting low level of population differentiation with significant amount of gene flow among populations (N_m_ = 2.125). This observation agrees well with corresponding values for woody perennial species with outcrossing or mixed mating system that maintain most of their variation within populations [63]. In *N. nimmoniana,* approximately 56% of total genetic variation was found within individuals followed by within populations (36%). This observation is similar to the values of ISSR marker based genetic variation detected in individuals distributed over a broad geographical area [64]. However, the genetic diversity levels detected in *N. nimmoniana* was higher than corresponding values reported in other microsatellite based analyses of natural populations of tropical tree species [49], [60]–[62], but similar to the values observed in *Milicia excelsa* [52]. However, historical rates of gene flow among populations estimated based on genetic structure can be confounded by several variables and must be interpreted with caution [65]–[67].

The species level inbreeding coefficient (F_IS_) indicates that alleles within populations were not united at random and that mating between close relatives may play an important role in determining the genetic structure of *N. nimmoniana*. At the population level, positive and significant F_IS_ values (Table 3) were detected in all eight populations examined. The significantly higher F_IS_ in NPA populations (Table 3) could be attributable to high harvesting pressure as expected in tree populations experiencing high level of harvesting and fragmentation leading to reduction in population size and the isolation of trees [54], [68], [69].

The results of Bayesian model based clustering method indicated geographical structure with clustering of populations into two major groups based on their geographical location. The neighbouring populations grouped together forming two separate and genetically distinct groups of populations (Fig. 5B and 5C). These results further suggest that genetic structuring of populations is low and weak. Several studies on spatial genetic structure of tropical and temperate species also reported that a weak or low genetic structure could be attributable to extensive gene flow among populations through seeds and pollen [69]–[71]. The structured distribution of two gene pools with predominant distribution of the gene pool 1 in the north and abundance of gene pool 2 in the South could be attributable to limited dispersal of seeds between north and south populations. In addition, several factors including habitat fragmentation and associated increase in selfing and inbreeding may also contribute to the genetic differentiation through genetic drift. Anthropogenic effects may also have contributed to the reduction of seed dispersal via birds leading to genetic structuring of *N. nimmoniana* [52], [69].

### Impact of harvesting on population structure

The size class structure of *N. nimmoniana* plants in PA and NPA populations were different, and PA populations maintained a higher proportion of larger, potentially older and reproductive plants than NPA populations. Furthermore we found a significant difference in density of adults and proportion of reproductive individuals in PA and NPA populations (P<0.05, Fig. 3B, C, D and E). Significantly, higher proportions of adults were harvested from NPAs as compared to PAs indicating that NPA populations suffer higher harvesting pressure as expected (Fig. 3F). This contributes to significant decline in demographic and reproductive parameters in NPA populations. Our results suggest that reproductive output changes with demographic shift in harvested populations. Populations with highest number of adults harvested had significantly low number of juveniles and saplings as compared to populations that were protected from harvesting. This effect could be attributable to shift in age class structure with harvesting, where non-protected populations continue to experience lower population sizes with fewer reproductive individuals leading to a decline in reproductive output and lower population growth rates.

Adults of *N. nimmoniana* in each population maintained significantly higher genetic diversity (A and H_O_) than the seedlings of the same population. This is because adult plants represent pre-harvesting generation with genetically diverse individuals resulting from larger effective population sizes. Recent human induced disturbance activities such as harvesting and fragmentation may have reduced the effective population sizes contributing to lowered genetic diversity over generations. Over harvesting leads to drastic reduction in population sizes and increased inbreeding, which can further reduce the genetic diversity in seedlings. This scenario was supported by the fact that there was significant increase in inbreeding values in seedlings as compared to adults in many populations (Table 3). Similar observations have been reported in several studies where selective logging and overharvesting of tropical tree species led to decrease in genetic diversity and increased inbreeding levels in seedlings [69], [72]. The altered age class distribution in NPA populations of *N. nimmoniana* due to harvesting may cause evolutionary effects compromising the long-term survival of the species. Documented changes in decline of demographic and reproductive parameters and genetic diversity measures in *N. nimmoniana* populations highlight the possible negative evolutionary consequences of harvesting. Although the short term demographic effects may be of immediate conservation importance [73], the selective harvesting of mature individuals may have profound long-term evolutionary impacts through the reduction of genetic diversity.

### Genetic diversity in protected and non-protected populations

This study indicates that populations in PAs harbor significantly higher level of genetic diversity (H_O_, A_P_, A_R_ and gene diversity) than NPA populations. Although other measures of genetic diversity (A and H_E_), did not differ significantly between two types of populations, the greater H_O_, A_P_, A_R_ and gene diversity indicates that allele frequencies are higher in populations in PA’s. These differences are due to difference in harvesting pressure between populations of PA and NPA. On average, more than 45% of adults were extracted from populations of NPA as opposed to less than 5% of adults extracted from PA. This change in demographic decline might have contributed to uneven distribution of alleles in NPA populations. We also observed that the genetic diversity parameters among adults and seedlings differed between populations of PA and NPAs. There was significant decrease in genetic diversity parameters (H_O_ and A) from adults to seedlings within populations of NPAs (Table 3), which is consistent with the general effects of harvesting of natural populations [18], [19]. As populations become small, rare alleles tend to be lost through the effect of genetic drift leading to the erosion of genetic diversity, which is often depicted in seedlings. Overall, our results demonstrate the impact of harvesting on genetic diversity of plants species and highlight the importance of PA network in conservation and management of economically and medicinally important plant species subject to harvesting [13].

### Population bottlenecks and predictions of future population sizes

The population bottleneck analyses using excess heterozygosity method (Sign test and Wilcoxon signed rank test) detected evidence of recent genetic bottlenecks in most of the *N. nimmoniana* populations sampled in the present study, but no difference between PA and NPA were found. The bottleneck analyses results were consistent among both adult and seedlings (Table 6). This suggests that irrespective of protected status of populations, *N. nimmoniana* populations experienced recent genetic bottlenecks, which is consistent with recent reports of demographic decline of *N. nimmoniana* [18], [19]. However, the allele frequency based analytical method did not reveal any signatures of bottleneck, suggesting that allele frequency based methods of population bottleneck analyses are not sensitive in detecting recent population bottlenecks. The excess in heterozygosity based methods are known to be more powerful in detecting recent genetic bottleneck signatures as compared to allele frequency distortion methods [46]. Occurrence of large number of rare alleles in a population may alter the distortion of allele frequency due to population bottlenecks and mask the genetic signature of recent bottleneck events. Our results revealed that most of the populations experienced significant decrease in heterozygosity parameters than observed number of alleles, reducing the sensitivity of detection of distortions in allele frequency distributions. The demographic data revealed a decline in *N. nimmoniana* populations in the wild, which is consistent with the detection of genetic bottlenecks in the present study. The reported 20% decline of *N. nimmoniana* in recent years may have contributed to reduction of effective population sizes throughout its distribution range leaving genetic signatures of population bottleneck. The excess heterozygosity method can detect population bottlenecks as recent as 6 to 120 years depending on the generation length of a given species [44], [74].

Our simulation study revealed a faster decline of observed number of alleles (OA) than H_O_ and is likely a result of existing low allelic diversity or recent genetic bottleneck. Similar results have been reported for other endangered species [75]. The BOTTLESIM simulation predicted a future decline in genetic diversity in most of the populations analysed (Fig. 6A to 6H). Interestingly, we observed that the current population size of *N. nimmoniana* in the Western Ghats is not sufficient to maintain present observed levels of genetic diversity over the period of next 100 years. This could be attributable to large scale harvesting of wild populations of *N. nimmoniana.* After the discovery of camptothecine in *N. nimmoniana*, the species have been largely exploited from the wild leading to reported 20% decline in natural populations of *N. nimmoniana* in the Western Ghats [18], [19]. The demographic decline coupled with recent genetic bottleneck events may contribute to further decline in genetic diversity in the future. Simulation results also predicted that populations of NPA lose diversity at a higher rate than populations from PAs. This may be due to variation in harvesting pressure, where over 45% of adults from NPAs have been harvested, but about only 5% of adults in PAs are known to have harvested. However, most of the populations are predicted to lose their diversity during next 100 years (Fig. 6A to 6H). Thus, measures to increase population sizes in PAs are needed to mitigate negative evolutionary consequences ensuring long term survival of *N. nimmoniana.*

### Conservation implications

Our results based on *N. nimmoniana* highlight the effectiveness of protected areas in conserving genetic diversity of economically and medicinally important plant species. We observed that the populations from PAs had significantly high genetic diversity than populations in NPAs. It was further supported by simulation analysis where NPAs are predicted to lose genetic diversity faster than PA populations in future. PAs were also effective in preventing harvesting pressure on populations as evidenced by harvesting of over 45% from NPAs as compared to about 5% adults harvested from PAs.

The long-term persistence of population depends on population size of a species. A population with large number of individuals is considered to have more genetic diversity, which increases their ability to adapt to changing environmental conditions [76]. On the other hand, reduction in population sizes leads to loss of genetic diversity and allelic richness, inbreeding and increased extinction risk [45]. In the present study, results of genetic bottleneck analysis showed that most of the populations have gone through a phase of recent genetic bottleneck indicating recent reduction in effective population size. The simulation analysis also indicated that the current population size of *N. nimmoniana* in Western Ghats is not sufficient to maintain 90% of present genetic diversity over the next 100 years. If population sizes of *N. nimmoniana* further continue to decline in the wild, most populations may lose nearly 50% of present genetic diversity during the next 100 years. The above results are consistent with recent demographic decline of *N. nimmoniana* in its natural habitat. The reduction in its habitat and population size may have led to genetic bottleneck and further loss in genetic diversity. The conservation strategy should be oriented towards protecting natural populations of *N. nimmoniana* in Western Ghats from further overexploitation to sustain the long-term survival of the species. As adults are known to maintain more genetic diversity, we recommend that conservation efforts geared toward protection of adult plants in each population should be implemented to protect the reproductive fitness and evolutionary potential of the species [77]. Overall these results highlight the need for establishing more protected areas (PAs) in Western Ghats to conserve genetic diversity of economically and medicinally important plant species.

## Supporting Information

File S1 **Supplementary Table. S1 Table.** Summary of t-test statistics for demographic parameters collected for four protected (PA) and non-protected (NPA) N. nimmoniana populations from Western Ghats.
(DOC)
