# Supplementary material for: Genetic Structure, Diversity and Long Term Viability of a Medicinal Plant, Nothapodytes nimmoniana Graham. (Icacinaceae), in Protected and Non-Protected Areas in the Western Ghats Biodiversity Hotspot
Source: PLoS One. 2014 Dec 10;9(12):e112769. doi: 10.1371/journal.pone.0112769 (PMC4262271; doi:10.1371/journal.pone.0112769)
Supplement: File S1 — Supplementary Table. S1 Table. Summary of t-test statistics for demographic parameters collected for four protected (PA) and non-protected (NPA) N. nimmoniana populations from Western Ghats. (DOC) [file pone.0112769.s001.doc]

| Density of adults/quadrat | | | | |
| --- | --- | --- | --- | --- |
|  | **DAN/JOI** | **KEM/VAD** | **AGU/MUL** | **TAL/BON** |
| **N** | 22/22 | 10/14 | 26/20 | 22/20 |
| **t-value** | 0.5589 | 1.8671 | 3.0883 | 3.3183 |
| **Df** | 42 | 22 | 44 | 40 |
| **p-value** | 0.579 | 0.0753 | 0.0035 | 0.0019 |
| **Mean** | 1.36/1.59 | 1/0.36 | 2.31/0.9 | 1.45/0.45 |
| **Standard error of mean (SEM)** | 0.22/0.34 | 0.33/0.17 | 0.34/0.27 | 0.23/0.18 |
| **Mean regenerants/adults** | | | | |
| **N** | 22/22 | 10/14 | 26/20 | 22/20 |
| **t-value** | 4.3581 | 4.9303 | 0.8478 | 2.9547 |
| **Df** | 42 | 22 | 44 | 40 |
| **p-value** | 0.0001 | 0.0001 | 0.4011 | 0.0052 |
| **Mean** | 2.735/0.409 | 5.8/0 | 0.729/0.400 | 1.435/0.200 |
| **Standard error of mean (SEM)** | 0.514/0.142 | 1.4/0 | 0.304/0.197 | 0.384/0.117 |
| **Mean regeneration/quadrat** | | | | |
| **N** | 22/22 | 10/14 | 26/20 | 22/20 |
| **t-value** | 4.7061 | 5.5341 | 1.8290 | 3.8385 |
| **Df** | 42 | 22 | 44 | 40 |
| **p-value** | 0.0001 | 0.0001 | 0.0742 | 0.0004 |
| **Mean** | 3.73/0.41 | 6.7/0 | 1.12/0.3 | 1.82/0.2 |
| **Standard error of mean (SEM)** | 0.69/0.14 | 1.45/0 | 0.37/0.18 | 0.39/0.12 |
| **Mean saplings/quadrat** | | | | |
| **N** | 22/22 | 10/14 | 26/20 | 22/20 |
| **t-value** | 2.7307 | 2.5355 | 2.6723 | 3.0708 |
| **Df** | 42 | 22 | 44 | 40 |
| **p-value** | 0.0092 | 0.0207 | 0.0038 | 0.0038 |
| **Mean** | 1.27/0 | 1/0 | 1.12/0 | 0.82/0.05 |
| **Standard error of mean (SEM)** | 0.47/0 | 0.39/0 | 0.37/0 | 0.23/0.05 |
| **% of stems harvested/quadrat** | | | | |
| **N** | 22/22 | 10/14 | 26/20 | 22/20 |
| **t-value** | 4.2322 | 9.4284 | 3.5963 | 4.8573 |
| **Df** | 42 | 22 | 44 | 40 |
| **p-value** | 0.0001 | 0.0001 | 0.0008 | 0.0001 |
| **Mean** | 0/40.325 | 0/82.632 | 11.154/53.333 | 4.583/32.500 |
| **Standard error of mean (SEM)** | 0/9.528 | 0/7.104 | 5.665/11.187 | 2.527/5.162 |

**Table S1.**
